# The Relationship Between Chest Imaging Findings and the Viral Load of COVID-19

**DOI:** 10.3389/fmed.2020.558539

**Published:** 2020-09-08

**Authors:** Wei Zhao, Lei He, Haoneng Tang, Xingzhi Xie, Lingli Tang, Jun Liu

**Affiliations:** ^1^Department of Radiology, The Second Xiangya Hospital, Central South University, Changsha, China; ^2^Department of Radiology, The First People's Hospital of Yueyang, Yueyang, China; ^3^Department of Laboratory Medicine, The Second Xiangya Hospital, Central South University, Changsha, China; ^4^Department of Radiology Quality Control Center, Changsha, China

**Keywords:** CT, COVID-19, viral load, follow-up, margin

## Abstract

**Purpose:** We aimed to investigate the relationship between clinical characteristics, radiographic features, and the viral load of patients with coronavirus disease 2019 (COVID-19).

**Methods and Materials:** We retrospectively collected 56 COVID-19 cases from two institutions in Hunan province, China. The basal clinical characteristics, detail imaging features and follow-up CT changes were evaluated and the relationship with the viral load was analyzed.

**Results:** GGO (48, 85.7%) and vascular enlargement (44, 78.6%) were the most frequent signs in COVID-19 patients. Of the lesions, 64.3% of the margins were uneasily differentiated. However, no significant correlations were found in terms of leucocytes, neutrophils, lymphocytes, platelets, and C-reactive protein (all *P* > 0.05). In contrast, the uneasily differentiated margin was negatively correlated with the *Ct* value (*r* = −0.283, *P* = 0.042), that is, an uneasily differentiated margin indicated a lower *Ct* value (*P* = 0.043). Patients with a lower *Ct* value were likely to present a progress follow-up change (*P* = 0.022). The *Ct* value at baseline could predict a progress follow-up change with an AUC of 0.685 (Cut-off value = 29.48). All four patients with normal CT findings presented new lesion(s) on follow-up CT scans.

**Conclusion:** The viral load of COVID-19 is negatively correlated with an uneasily differentiated lesion margin on initial CT scan images and the *Ct* value should noted when making a diagnosis. In addition, following-up CT scans are necessary for patients who presented a normal CT at the initial diagnosis, especially for those with a low *Ct* value.

## Key Results

- The uneasily differentiated margin was negatively correlated with the *Ct* value (*r* = −0.283, *P* = 0.042). In another words, an uneasily differentiated margin indicated a lower *Ct* value.
- Follow-up CT scans are necessary for patients with normal CT findings at initial diagnosis, especially for those with a low *Ct* value.

## Introduction

A cluster of “unknown viral pneumonia” cases in Wuhan, China, was reported to World Health Organization (WHO) on December 31 2019 (1). A novel coronavirus, named severe acute respiratory syndrome coronavirus 2 (SARS-CoV-2) was identified through deep sequencing analysis (2). The outbreak of coronavirus disease 2019 (COVID-19), declared as a public health emergency of international concern (PHEIC) (3), has raised intense concerns around the world (1). The situation of the outbreak of COVID-19 in China has been brought under control (4), however, it still threatens the global medical system.

The genome sequence findings suggested that the presence of COVID-19 was closely related to another coronavirus termed severe acute respiratory syndrome (SARS)-related CoV (5). According to the latest study (6), the modality of COVID-19 is lower than that of SARS-CoV. It has been proven that the possibility of human-to human transmission (7, 8) and the R_0_ (*i*.e., the expected number of additional cases that one case will generate) ranges from 2 to 3 (9). Since the pathogenesis and the many comprehensive biological features (*i.e*., the microenvironment change and immune system reaction) of COVID-19 remain undiscovered, no specific antiviral agent and effective vaccine is available for treatment of this disease (10). Early detection, early diagnosis, early isolation, and early therapy remain the basic and essential strategies (11). Accurately assessing the disease severity of COVID-19 is still vital for clinical treatment scenarios and taking action in advance to avoid the presence of rapid progress. The viral load, inversely correlated with the cycle threshold (*Ct*) value, is considered as a parameter to reflect the disease severity (12–14) and indicate the transmission ability (15). However, not all hospitals reported the *Ct* value and only gave a binary diagnosis (*i.e.*, positive or negative). Moreover, the assessment of the *Ct* value of the virus needs a real-time reverse transcription-polymerase chain reaction test (RT-PCR), which has inherent disadvantages including possible false positive results and a long turnaround time. Identifying the potential clinical alternative factors of the *Ct* value may help us assess the disease severity efficiently.

Several available clinical factors, such as white blood cell/neutrophil/lymphocyte count, might have the potential to reflect the severity of COVID-19 (6, 8, 16). The clinical importance of computed tomography (CT) is emphasized by the evidence of its value in the screening, diagnosis, and evaluation for the daily treatment of patients with COVID-19 in clinical practice (17–19). Moreover, the radiographic features are also reported to reflect the severity of COVID-19 (17–19). Therefore, all the aforementioned potential risk factors may throw light on the viral load indirectly and be considered as convenient and alternative factors to reflect the condition of COVID-19. However, the relationship between the aforementioned risk factors with viral load remains unclear.

In the present research, the purpose is to investigate the relationship between clinical characteristics, radiographic features, and *Ct* values in patients with COVID-19 and provide some hints for its early diagnosis.

## Materials and Methods

This retrospective study was approved by our Medical Ethical Committee (Approved Number. 2020002), which waived the requirement for patients' informed consent referring to the CIOMS guideline.

### Patients

In the study, we retrospectively included confirmed COVID-19 cases from Hunan Province, China. From January 16 2020 to February 6 2020, a search of the electronic system and the picture achieving and communication system (PACS) was performed to collect clinical features, laboratory values (the first one upon admission), epidemic characteristics, and all scanned CT images. The inclusion criteria included: (1) patients with PCR-confirmed COVID-19; (2) patients who underwent CT scanning before treatment; (3) the interval between a CT scan and throat swab sample being taken was <2 days; (4) the initial viral load was reported. The exclusion criteria included: (1) patients without PCR-confirmed COVID-19; (2) patients that had not undergone CT scanning before treatment; (3) the interval between a CT scan and throat swab sample being taken was more than 3 days; (4) No viral load was reported. Finally, 56 of 360 cases (30 women, 26 men; mean age, 50.34 years ± 15.65 [SD]; age range, 2–79 years) were included (27 patients from the Second Xiangya Hospital and 29 patients from the First People's Hospital of Yueyang). We characterized patients into four groups, mild type, common type, severe type, and fatal type based on the guideline of COVID-19 (Trial Version 7) (20), proposed by the China National Health Commission. Based on the different treatment regimens, we divided the included patients into two groups, non-severe group (mild type and common type) and severe group (severe type or fatal type). The interval between the onset of the disease and CT scans was 5 (2–8), presented as the median (Inter quartile range).

### PCR Method

Duplex RT-PCR assays were performed by using throat swab samples in accordance with the protocol established by WHO (21). The nucleic acid was extracted by using an automatic system (Nathch CS, sansure biotich, Hunan). The nucleic acid amplification was performed on slan96P (Shanghai Hongshi Medical Technology Co., LTD). Each reaction tube was internally controlled. The *Ct* value was recorded for all samples and a *Ct* value <40 and >0 was considered as PCR positive.

### Imaging Technique and Imaging Interpretation

All CT scans were performed with the following three scanners: Somatom definition AS (Siemens Medical Solutions), Somatom emotion (Siemens Medical Solutions), and ANATOM 16HD (ANKE Medical Solutions). The acquisition parameters were as follows: 120 kVp; 100–200 mAs; pitch, 0.75–1.5; and collimation, 1–5 mm, respectively. All imaging data were reconstructed by using a medium sharp reconstruction algorithm with a thickness of 1 mm. CT images were acquired in the supine position at full inspiration for all patients. All chest CT scans were reviewed blindly and independently by two radiologists (with 5 and 15 years of experience). If an inter-observer difference happened, the two radiologists would re-review the imaging feature(s) together and reach an agreement (in consensus). All images were viewed on both lung (width, 1,500 HU; level, −700 HU) and mediastinal (width, 350 HU; level, 40 HU) settings. Twelve imaging features including features of ground-glass opacities (GGO), consolidation, mixed GGO and consolidation, margin of the lesion (easily differentiated and uneasily differentiated, based on the lesions-lung interface), architectural distortion, reticulation, traction bronchiectasis, sub-pleural bands, intrathoracic lymph node enlargement, fibrosis, vascular enlargement in the lesion, and pleural effusions were evaluated according to our previous studies (18, 22). The number of involved lung lobes, the craniocaudal distribution (upper lung predominant, lower predominant, and no craniocaudal distribution), the transverse distribution (central or peripheral or no transverse distribution), and the scattering distribution (focal, multifocal, or diffuse) were also evaluated. The transverse distribution of the abnormalities were categorized as central (i.e., peribronchovascular), peripheral (i.e., sub-pleural), or with no transverse predilection. Focal was defined as a single lesion of abnormality, multifocal as more than one lesions, and diffuse as involvement of most of the volume of one lung lobe. A CT score system was used to evaluate the extent of disease (23). We defined three imaging changes: no change, progress change, and improvement change (22). No change referred to no obvious changes presented in the chest CT. Progress change referred to the presence of new lesions or the presence of an extent involvement area during the treatment. Improvement change referred to continually absorbed abnormities.

### Statistical Analysis

Continuous variables were presented as median (IOR) and categorical variables were presented as numbers (%). The correlations between clinical features, laboratory tests, imaging features, and viral load were analyzed using the Spearman analysis. The ROC analysis was used to investigate the performance of the *Ct* value in predicting the follow-up change. A two-sided *P* < 0.05 was considered statistically significant. All statistical analyses were performed using the SPSS software (version 24.0).

## Results

### Clinical Characteristics and Laboratory Detection

In the beginning, 4 patients (male 1, female 3) were divided into the mild group, 49 patients (male 23, female 26) were common, another 3 patients (male 2, female 1) were in the severe group. Twenty-one (37.5%) patients had a direct exposure history link to Wuhan (*i.e.*, long-term exposure history to Wuhan, traveling in Wuhan before diagnosis), 37 (66.1%) patients had an exposure history to confirmed patients. It is noted that 4 (7.1%) patients denied any direct exposure history and indirect exposure to confirmed patients and 17 (30.3%) patients were related to a family outbreak (more than 2 patients were confirmed in one family). Fever (36 of 56, 64.3%) and cough (31 of 56, 55.4%) were the most common onset symptoms. Other onset symptoms, including myalgia or fatigue, sore throat, dyspnea, diarrhea, nausea, and vomiting were presented in Tables 1, 2 patients had no onset symptoms and most patients (80.4%) had no underlying disease. The information about laboratory tests are also presented in Table 1. The median *Ct* value was 33.20 in our cohort.

**Table 1:** Clinical features, laboratory tests in our cohort.

**Basal characteristics**	**All patients (*n* = 56)**
**Sex**	
Male	26
Female	30
Age (years)^a^	50.34 ± 15.65
**Epidemic history**	
Direct exposure history^b^	21 (37.5)
Indirect exposure history	37 (66.1)
No exposure history	4 (7.1)
Family outbreak	17 (30.3)
**Onset symptoms**	
Fever	36 (64.3)
Cough	31 (55.4)
Myalgia or fatigue	10 (17.9)
Sore throat	6 (10.7)
Headache	5 (8.9)
Dyspnea	4 (7.1)
Diarrhea	2 (3.6)
Nausea and vomiting	1 (1.8)
More than one symptom	35 (62.5)
None	2 (2)
**Underlying disease**	
Cardiovascular and cerebrovascular diseases	3 (5.4)
Surgery history	1 (1.8)
Digestive system disease	3 (5.4)
Respiratory system disease	3 (5.4)
Endocrine system disease	2 (3.6)
None	45 (80.4)
Leucocytes (× 10^9^ per L)^c^	4.83 (3.57–6.24)
Neutrophils (× 10^9^ per L)	2.92 (2.35–4.18)
Lymphocytes (× 10^9^ per L)	1.08 (0.77–1.43)
Platelets (× 10^9^ per L)	163.50 (121–202)
C-reactive protein (mg/L)	14.95 (4.22–39.04)
*Ct* value	33.20 (28.30–36.66)

a *presented as mean ± SD*,

b *presented as number (percentage)*,

c *presented as median (Inter quartile range)*.

**Table 2:** Imaging finds of patients with COVID-19.

**Imaging findings**	**All patients (*n* = 56)**
GGO	48 (85.7)
Vascular enlargement	44 (78.6)
Margin (uneasily differentiated)	37 (66.1)
Reticulation	26 (46.4)
Traction bronchiectasis	26 (46.4)
Consolidation	24 (42.9)
Fibrosis	22 (39.3)
Mixed GGO and consolidation	21 (37.5)
Architectural distortion	18 (32.1)
Sub-pleural bands	13 (23.2)
Pleural effusions	1 (1.8)
Intrathoracic lymph node enlargement	0 (0)
**Craniocaudal distribution**	
Upper lung predominant	6 (10.7)
Lower lung predominant	19 (33.9)
No craniocaudal distribution	27 (48.2)
**Transverse distribution**	
Central	0 (0)
Peripheral	46 (82.1)
No transverse distribution	6 (10.7)
**Scattering distribution**	
Focal	2 (3.6)
Multifocal	32 (57.1)
Diffuse	18 (32.1)
CT score	6 (3–7.75)
**Number of involved lung lobe**	
0	4 (7.1)
1	2 (3.6)
2	6 (10.7)
3	6 (10.7)
4	16 (28.6)
5	22 (39.3)
**Number of lesions**	
>5	40 (71.4)
<5	16 (28.6)
Number of absent CT findings	4 (7.1)
**Follow-up CT changes**	
Improvement change	27 (51.8)
Progress change	25 (41.1)

*Data were presented as numbers (percentage), except for CT score, which presented as median (Inter quartile range)*.

### CT Findings

**Figure 1:**
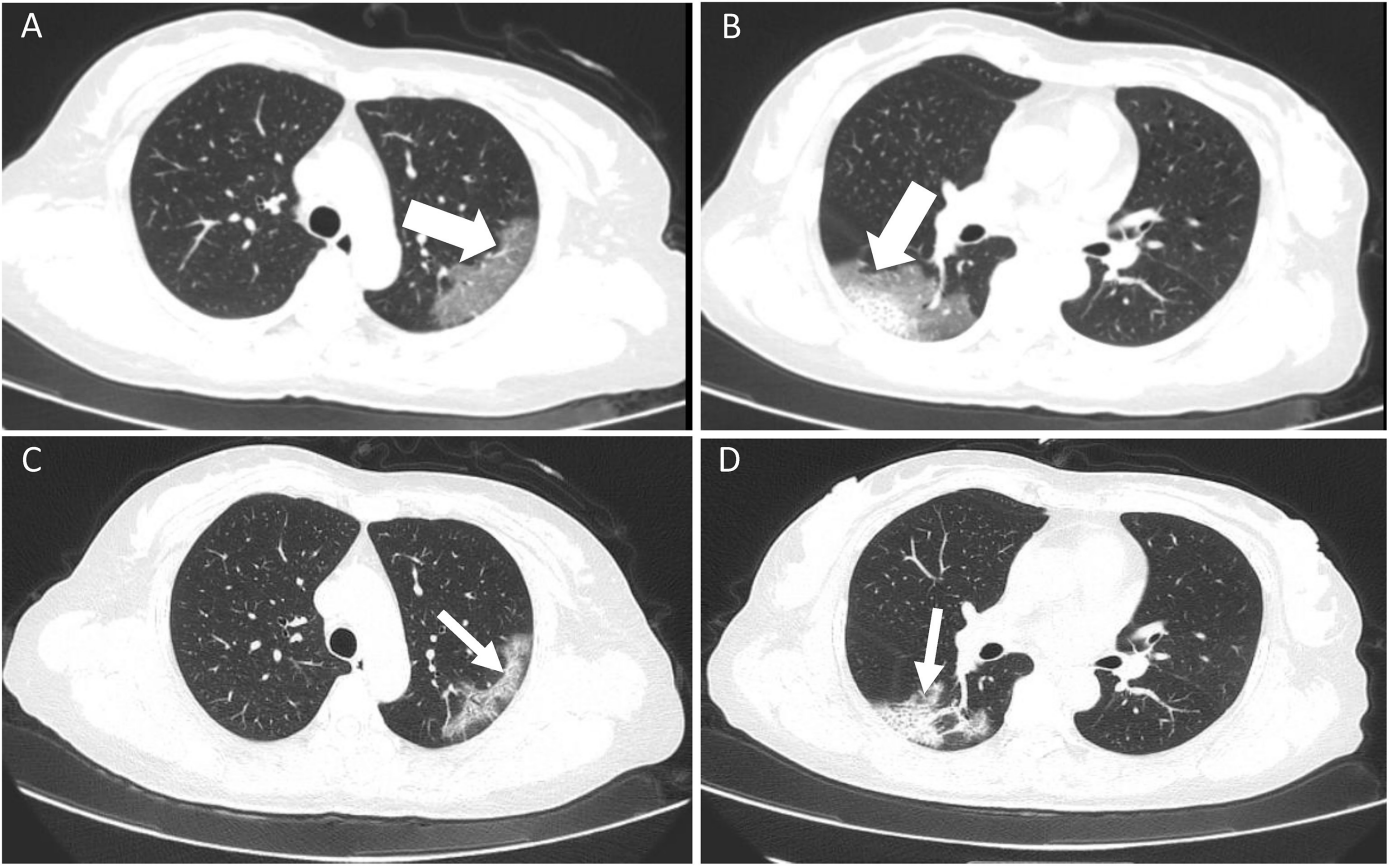
A 52-year old female with confirmed COVID-19 infection. Patient had close contact with a confirmed case and the onset symptom of fever. **(A,B)** Initial CT scan (performed on February 5 2020) showed bilateral GGO and mixed GGO and consolidation (white thick arrow) with an easily differentiated margin. The viral load (*Ct* value) was 38.65. **(C,D)** The follow-up CT scan (performed on February 9 2020) showed an improvement change. All the lesions had been absorbed (white fine arrow).

**Figure 2:**
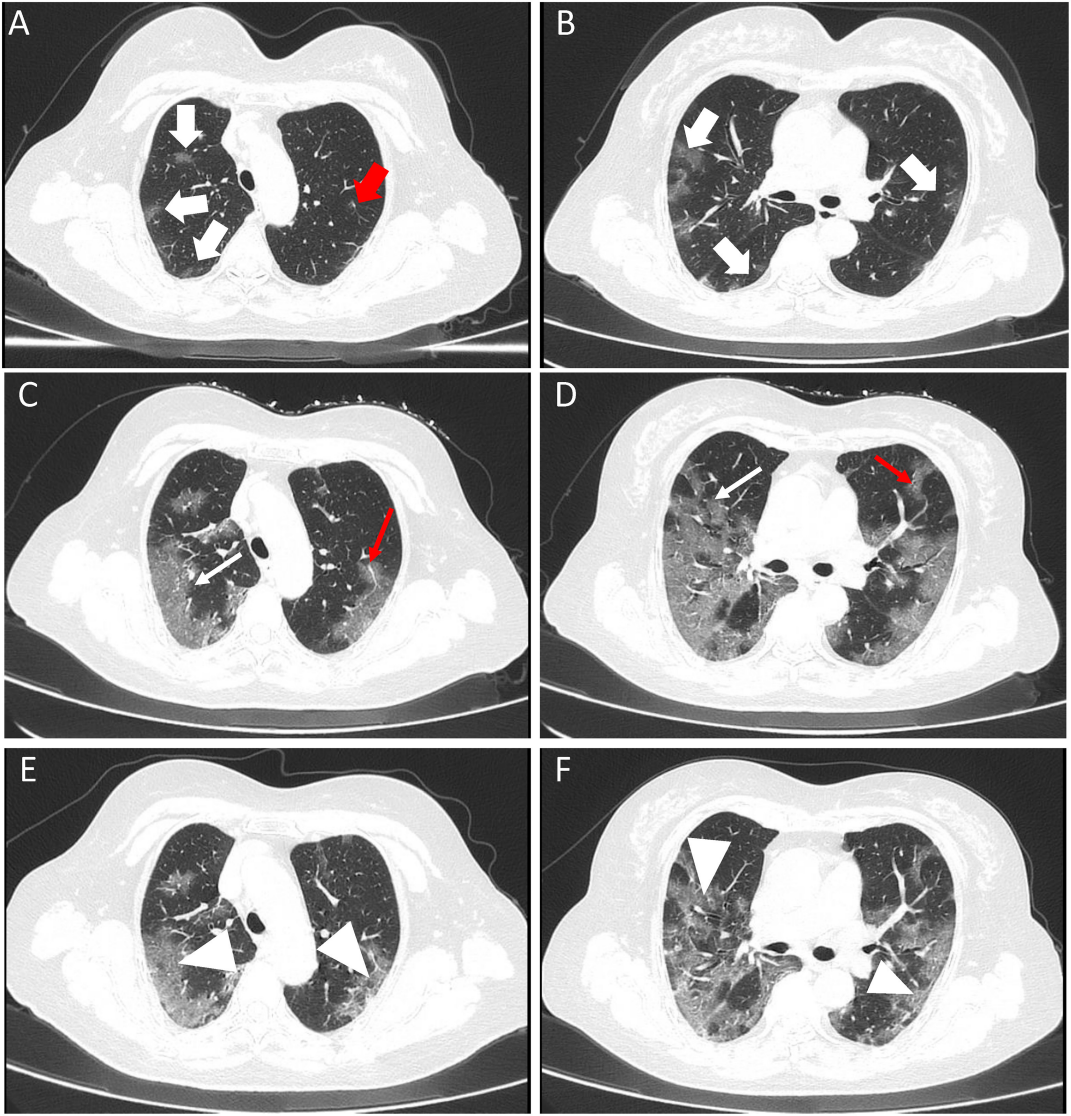
A 67-year old female with confirmed COVID-19 infection. Patient had close contact with a confirmed case and the onset symptom of fever. **(A,B)** Initial CT scan (performed on January 31 2020) showed bilateral GGOs (white thick arrow) with an uneasily differentiated margin. The viral load (*Ct* value) was 29.13. **(C,D)** The first follow-up CT scan (performed on February 5 2020) showed a progress change. All the lesions had been enlarged (white fine arrow). The diameter of the vascular was larger than that of initial CT image [red fine arrow of **(C)**] and a new lesion was presented [red fine arrow of **(D)**]. Please note that the margin of lesions on first follow-up CT images was clearer than before. The second follow-up CT scan (performed on February 8 2020) showed an improvement change (white arrowhead) **(E,F)**.

GGO (48 of 56, 85.7%) and vascular enlargement (44 of 56, 78.6%) were the most frequent signs in COVID-19 patients (Figures 1, 2). The lesion's margins were 64.3% uneasily differentiated. Intrathoracic lymph node enlargement and pleural effusions were rare findings in our cohort. Lesions were more likely to be peripherally distributed (46 of 56, 82.1%) and contain bilateral involvement (49 of 56, 87.5%). 39.3% of patients had 5 lung lobes involved and 71.4% of patients had more than 5 lesions. Other evaluated imaging features are described in detail in Table 2. The median CT score of the lung involvement was 6. It is notable that 4 patients had no obvious abnormity on initial CT images.

### The Relationships Between Clinical Factors, Imaging Findings and *Ct* Value

We investigated the relationships between clinical factors, imaging findings, and *Ct* value. No significant correlations were found in terms of leucocytes, neutrophils, lymphocytes, platelets, and C-reactive protein (all *P* > 0.05). In contrast, the uneasily differentiated margin was negatively correlated with the *Ct* value (*r* = −0.298, *P* = 0.026, Table 3), that is, an uneasily differentiated margin indicated a lower *Ct* value, which potentially indicated a more severe presentation of the disease (Figures 1, 2).

**Table 3:** The correlations between clinical features, laboratory tests and imaging features and viral load.

**Characteristics**	***Ct*** **value**	
	***r***	***P***
Sex	0.181	0.183
Age	0.086	0.529
Leucocytes (× 10^9^ per L)	0.087	0.521
Neutrophils (× 10^9^ per L)	0.148	0.277
Lymphocytes (× 10^9^ per L)	−0.071	0.603
Platelets (× 10^9^ per L)	0.058	0.672
C-reactive protein (mg/L)	0.096	0.483
GGO	0.208	0.123
Vascular enlargement	0.094	0.490
Margin (reference: easily differentiated)	−0.298	**0.026**
Reticulation	−0.024	0.859
Traction bronchiectasis	−0.095	0.485
Consolidation	0.051	0.707
Fibrosis	0.152	0.265
Mixed GGO and consolidation	0.01	0.94
Architectural distortion	−0.05	0.716
Sub-pleural bands	−0.071	0.605
Pleural effusions	0.104	0.444
Craniocaudal distribution	0.158	0.246
Transverse distribution	0.081	0.555
Scattering distribution	0.135	0.322
CT score	0.179	0.187
Number of involved lung lobe	0.256	0.057
Number of lesions	0.125	0.360
Number of absent CT findings	0.227	0.092
Follow-up CT changes (reference: improvement change)	−0.322	**0.016**

*The bold numbers indicated an significant correlation*.

### Follow-Up CTs and The Relationship With *Ct* Value

**Figure 3:**
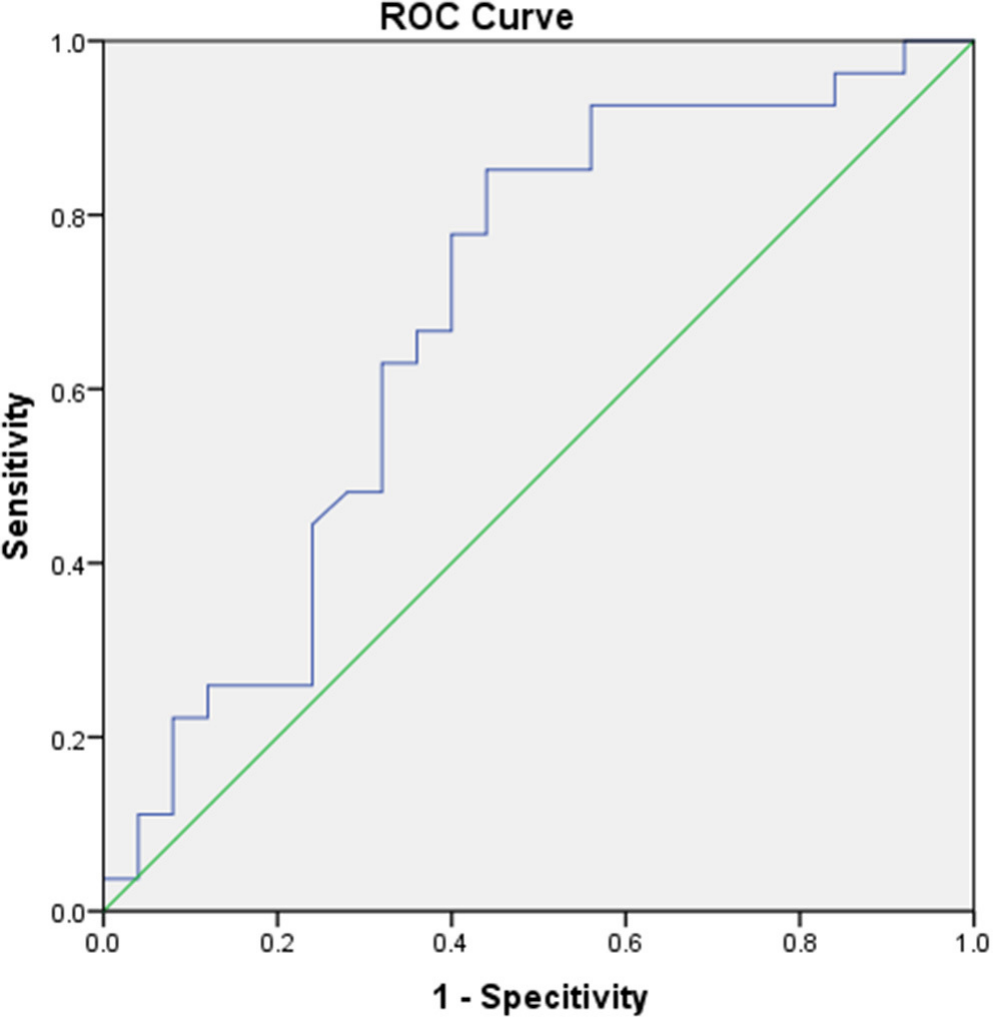
The ROC curve of Ct value in predicting the follow-up CT changes.

**Figure 4:**
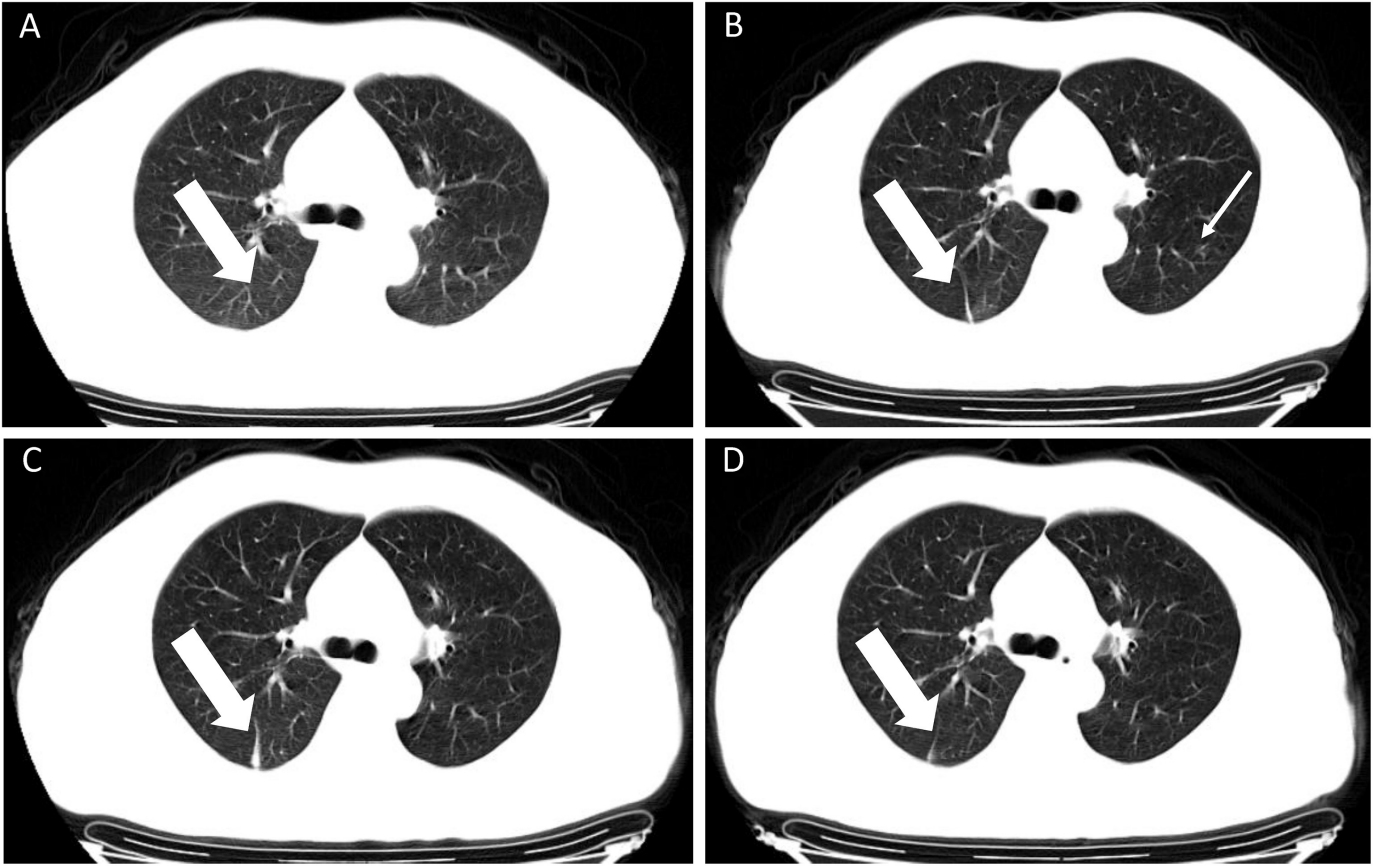
A 45-year old male with confirmed COVID-19 infection. Patient had a direct exposure history to Wuhan and the onset symptom of vomiting. **(A–D)** CT scan performed four times. Initial CT scan (performed on January 30 2020) showed no obvious abnormal CT findings **(A)**. The next three times follow-up CT images showed a strip lesion in the right lower lobe (white thick arrow), first presented on second CT scan [performed on February 3 2020, **(B)**], enlarged on the third CT scan [performed on February 6 2020, **(C)**] and absorbed on the fourth CT scan [performed on February 9 2020, **(D)**]. An ambiguous lesion was shown in the upper left lobe [white fine arrow, **(B)**] and was absent in other images. The viral load (*Ct* value) was 22.53.

In total, 52 of 56 (92.9%) patients had undergone follow-up CT scans. Among the 52 patients, 27 patients presented an improved change, whereas 25 patients presented a progressed change. Furthermore, we investigated the relationships between the follow-up CT changes and *Ct* value. The results showed that the progressed follow-up change was negatively correlated with the *Ct* value (*r* = −0.322, *P* = 0.016, Table 3), that is, patients with a lower *Ct* value were likely to present a progressed follow-up change (*P* = 0.022). The *Ct* value at baseline could predict a progress follow-up change with an AUC of 0.685 (Cut-off value = 29.48) (Figure 3). All 4 of the patients (*Ct* value: 25.23, 29.37, 25.22, and 33.19, respectively), with normal CT findings presented new lesion(s) on follow-up CT scans (Figure 4).

## Discussion

In the present study, we investigated the relationships between clinical characteristics, radiographic features, and *Ct* values in patients with COVID-19 and we found that an uneasily differentiated margin of lung lesions was negatively correlated with the *Ct* value, which could be used as a predictor for the severity of COVID-19, that is, patients with a lower *Ct* value were likely to present a progress follow-up change (*P* = 0.022).

Both the number of confirmed cases and deaths has overtaken that of SARS in China (24). The clinical features and epidemic history have been well-reported recently. The onset symptom of fever and specific exposure history were also reported in our study. Most patients (66.1%) had an indirect exposure history and 17 (30.3%) patients were related to a family outbreak. The incidence indicated a serious risk of human-to-human transmission, therefore, early identification of positive cases and separating the negative patients from the suspected group is urgently warranted.

Although advances in treatment scenarios have been made, there is no existing evidence of curative medicine for COVID-19. Early diagnosis and treatment remained the basic strategies. The treatment response and clinical outcome of patients with COVID-19 were not well-documented, especially for severe/fatal patients or patients with rapid progress, so identifying patients with potential rapid progress early, accurately evaluating the severity of the disease at baseline, and further predicting clinical outcomes may improve the prognosis and curative rate. It was reported that the viral load (*Ct* value) has the potential to determine the severity of the disease (14). However, obtaining the viral load needs a long-term PCR test which has the potential of providing a false negative (18), therefore, investigating the relationships between these factors and the vial load may overcome the disadvantage. Leukopenia, lymphopenia, thrombocytopenia, and elevated C-reactive protein (CRP) levels were identified as risk factors for severe cases (6, 8). Liu et al. has discovered that the *Ct* value of the virus highly correlates with CRP and lymphopenia in patients with COVID-19. However, no laboratory manifestations were correlated with the viral load which may contribute to data bias, given the fact that we included a relatively large sample size.

CT scans, most frequently used in the diagnosis and monitoring treatment response of COVID-19, has contributed a lot in clinical practice. The typical chest CT features have been reported in previous studies (17, 25). GGO was the most frequent sign among the positive patients in our study, which is consistent with previous studies (25). In addition, the radiographic features were also considered as predictors for the severity of the disease (6). The CT score, a semi-quantitative score to evaluate the extent of the lesions, was a severity predictor in our previous study (26). However, it had no statistical correlation with the viral load. Moreover, another factor related to the extent of the lesions, e.g., number of involved lung lobe and the number of lesions were also not significantly correlated with the viral load. A low viral load may cause more serious reactions in the body, leading to a higher extent of lesions in the lung. The unexpected results may be due to the small sample size. Although the lesions were more likely be peripherally distributed and multifocal, the viral load had no predominant distributions. In other words, the distributions can be considered as a differentiated feature from other viral-related pneumonia instead of a severity predictor. Interestingly, we found that an uneasily differentiated margin indicated a lower *Ct* value, which possibly indicated the severity of the disease. The suggestion that an uneasily differentiated margin could indicate the reaction of the immune system against COVID-19 is still ongoing and the potential of further progress is expected. In contrast, an easily differentiated margin indicates that the virus has been restricted.

We also found the follow-up CT changes could help identify the patients who might progress in the later stage in our previous study (22). In this study, we also investigated the relationship between the viral load and the follow-up CT changes and found that the progressed follow-up changes were negatively correlated with the *Ct* value, which means patients with a lower *Ct* value are likely to present a progressed follow-up CT change, maybe even a worse prognosis. The *Ct* value at baseline yields an AUC of 0.685 to predict a progress follow-up CT change.

It is notable that 4 patients had no abnormal CT findings in our cohort. All 4 of the patients presented new lesions in the follow-up CT scan images, indicating that abnormal imaging findings might be absent in the early stage of COVID-19. This also further proved a lower *Ct* value are likely to present a progress follow-up CT change. It reminds physicians of the importance of follow-up CT scans for patients with normal CT findings at initial diagnosis, especially for those with a low *Ct* value.

Nevertheless, the study has several limitations. Firstly, this is the experience of a single center and the sample size was small. Our conclusions cannot be generalized to other centers taking care of COVID-19 patients directly, which needs further investigation. A multicenter study and/or including more cases might provide more information on the viral load and clinical outcomes of COVID-19. Secondly, the relationship between *Ct* value during the treatment and clinical features, laboratory tests and radiographic features were not investigated and will be conducted in a future study.

In conclusion, the viral load is negatively correlated with an uneasily differentiated lesion margin on initial CT scan images and the Ct value should be paid attention to in making the diagnosis. In addition, follow-up CT scans are necessary for patients with normal CT findings at initial diagnosis, especially for those with a low *Ct* value.

## Data Availability Statement

The original contributions presented in the study are included in the article/supplementary material, further inquiries can be directed to the corresponding author/s.

## Ethics Statement

The studies involving human participants were reviewed and approved by Medical Ethical Committee. Written informed consent for participation was not provided by the participants' legal guardians/next of kin because: This was a retrospective study.

## Author Contributions

JL, WZ, and LT: conception and design. JL and LT: administrative support. JL, WZ, and LH: provision of study materials or patients. JL, WZ, LH, HT, and XX: collection, assembly of data, data analysis, and interpretation. All authors: manuscript writing and final approval of manuscript.

## Conflict of Interest

The authors declare that the research was conducted in the absence of any commercial or financial relationships that could be construed as a potential conflict of interest.
